# Potential Effects of the COVID-19 Pandemic on Future Birth Rate

**DOI:** 10.3389/fpubh.2020.578438

**Published:** 2020-12-10

**Authors:** Md. Asad Ullah, Abu Tayab Moin, Yusha Araf, Atiqur Rahman Bhuiyan, Mark D. Griffiths, David Gozal

**Affiliations:** ^1^Department of Biotechnology and Genetic Engineering, Faculty of Biological Sciences, Jahangirnagar University, Dhaka, Bangladesh; ^2^Department of Genetic Engineering and Biotechnology, Faculty of Biological Sciences, University of Chittagong, Chattogram, Bangladesh; ^3^Department of Genetic Engineering and Biotechnology, School of Life Sciences, Shahjalal University of Science and Technology, Sylhet, Bangladesh; ^4^Department of Microbiology, Faculty of Biological Sciences, University of Chittagong, Chattogram, Bangladesh; ^5^International Gaming Research Unit, Psychology Department, Nottingham Trent University, Nottingham, United Kingdom; ^6^Department of Child Health, MU Women's and Children's Hospital, University of Missouri School of Medicine, Columbia, MO, United States

**Keywords:** COVID-19, pandemic, demography, birth rate, fertility

## Abstract

Here, we examine the potential effect of the COVID-19 pandemic on future birth rates. This highly contagious disease originated in China, and rapidly spread worldwide, leading to extensive lockdown policies being implemented globally with the aim of containing the infection rates and its serious attendant consequences. Based on previous extant literature, this paper overviews the potential demographic consequences of the current progressively widespread epidemic on conception and fertility as driven by the data obtained during similar prior incidents. In general, epidemics manifest a common pattern as far as their impact on population, which is remarkably similar to natural disasters, i.e., a steep decline in birth rates followed by gradual increases and then followed by a baby boom. Additionally, we have also depicted how economic conditions, mental health, fear, and mortality may also influence future birth rates.

## Introduction

The coronavirus disease-2019 (COVID-19) is an infectious disease caused by Severe Acute Respiratory Syndrome Coronavirus 2 (SARS-CoV-2), which was first reported in December 2019 in Wuhan, People's Republic of China. To date, the spread of this disease has been extremely rapid throughout the world (1), and the World Health Organization (WHO) declared COVID-19 as a Public Health Emergency on January 30, 2020. By March 11, 2020, COVID-19 was declared as a global pandemic by the WHO, and has since continued to spread at an accelerated rate. At the time of writing, more than 50.5 million cases have been reported across 213 countries and territories, and have ultimately resulted in more than 1.26 million deaths (November 9, 2020; (Source: Johns Hopkins University Coronavirus Resource Center; https://coronavirus.jhu.edu/map.html). The virus generally spreads between persons during close contact, most often through small droplets produced by sneezing, coughing, and talking. It can also spread by touching contaminated surfaces followed the touching of the face, nose, and eyes with unwashed hands. Since no licensed vaccines or specific antiviral treatments are currently available for COVID-19, some initiatives such as spatial distancing restrictions and lockdowns across the world have been strictly imposed to prevent the spread of the virus and reduce the magnitude of the pandemic.

As a result of the transmission control efforts, more than two-thirds of the world population have experienced lockdown measures, lasting from weeks to months, and thereby affecting family and social lives, as well as imposing a substantial burden on mental health (2). Thus, in addition to the physical health effects of the virus in those persons infected, the pandemic is also causing detrimental social and mental health effects, which in turn can influence fertility, conception, gestation, and birth. Furthermore, different propagation patterns of the COVID-19 pandemic as occurring in different countries and even in regions within countries may also in turn lead to other consequences, the latter related to different socio-economic conditions, healthcare facilities and access, and financial stability (2). Thus, the impact of the pandemic on conception, pregnancy, and birth will likely greatly differ in advanced and emerging economies.

## Influences of COVID-19 Pandemic on Fertility Rate Worldwide

### The Socio-Economic Impact of the COVID-19 Pandemic

The impact of the lockdown may vary from country to country, and it is likely to increase global poverty and inequalities (3–6). Millions of individuals are unable to work because of complete or partial lockdown, and unemployment rates have exponentially risen. Consequently, individuals from all walks of life have been afflicted by the financial fluctuations and economic uncertainty during the outbreak, and the situation has led to economic recession and increased psychological stress (2). The World Bank projects that the COVID-19 pandemic will cause a contraction of 7% in GDP across the globe in 2020 and, while severely affecting all countries, the impact on unemployment will vary. According to estimates by the International Labor Organization (ILO), the lower-middle income countries (LMICs) (16.1%) experienced greater levels of working hour losses than those sustained by higher income countries (HICs) (13.9%) in the second quarter of 2020.

### The Impact of Socio-Economic Circumstances on Fertility Rates

Studies suggest that fertility rates are affected by economic recession and poverty (7) with country-specific poverty rates across both emerging and developed economies leading to further variation in fertility rates (8). USA experienced a decline in birth rates during the great economic recession in 2008, and the trend was sustained till the first half of 2009, whereas the birth rates in 2007 were the highest recorded for the prior two decades. A study carried out by Pew Research Center in October 2009 in USA reported that 14% (ages 18–34) and 8% (ages 35–44) of those surveyed were still planning to postpone having a child due to the prior financial downturn (9). Other factors such as the availability of contraception, and women educational attainment levels (9) may also influence the fertility rates differently across HICs and LMICs. Therefore, the economic recession caused by the COVID-19 pandemic may impose a long-term impact on fertility rate, even after the pandemic has abated or been resolved.

### The Impact of Anti-COVID-19 Measures on Fertility Rates in HICs

The economic crisis that resulted from COVID-19 pandemic along with unemployment, increase in domestic violence, and limited access to the healthcare sector in the antenatal period can also affect birth rates. The United Nations predicted that in 114 LMIC, 47 million women will be unable to access modern contraceptives due to lockdown measures and will lead to 116 million unwanted babies. In addition, 3.3 million unintended pregnancies are estimated in the USA. However, this unwanted baby boom will not be a major concern for developed economies according to the United Nations.

Fertility is usually reported as the Total Fertility Rate (TFR), indicative of the average number of children per woman. A TFR of ~2.1 children per woman is termed the Replacement Fertility Rate. The TFR has been below the replacement rate in most developed countries since 1950. The unique circumstances imposed by the pandemic are likely to affect TFR, particularly in developed economies, where the population susceptibility to economic changes appears to exert increased impact on reproductive decisions (10, 11). Moreover, in HICs the fertility rate is greatly influenced by higher women educational levels, which again may impact the birth rates in high economies during COVID-19 pandemic (12). The desire to conceive a baby is also somewhat dependent on the childcare outsourcing in HICs, and fertility is also maintained in this way. The inaccessibility to childcare outsourcing services during COVID-19 pandemic could also impact the birth rates to some extent in higher socioeconomic settings (12).

According to a survey among ovulation and pregnancy test kit customers (*n* = 132) in the USA, many couples have expressed reluctance to conceive babies in such adversity (13). The survey also reported that the supply of ovulation and pregnancy test kits decreased and the demand of emergency contraception increased in May 2020, whereas, there was a spike in the demand of pregnancy and ovulation test kits early in March 2020 (13) suggesting that less individuals are trying to become pregnant. Though the survey in the USA, which included only a limited number of participants, may not accurately represent all the actual scenarios, another survey in Italy involving highly educated participants (~64% graduate) found that most of them were not planning to conceive during the COVID-19 crisis (14). Their sex lives as well as planning for parenthood have been substantially influenced during COVID-19 pandemic (14) by a number of reasons like worries about future economic difficulties, fear of getting infected, complications during pregnancy, shortage of healthcare workers, and disease clusters in hospitals. Conversely, a minority of individuals may be more inclined to conceive during the lockdown (14). This is likely due to enhanced couple intimacy opportunities in the context of working from home or furlough during lockdown, emergent desire to bring about a change in their life, and the need for positive emotional support during the COVID-19 pandemic (15). Again, the study also revealed that the desire for parenthood during the pandemic was more prevalent among the higher age group (31–46 years) which may be another reason for the fluctuation of fertility rates.

The aforementioned study concerning family planning among 1,482 Italian respondents (944 males and 538 females) reported that, before the COVID-19 pandemic, 268 participants were planning to have children and the other 1,214 showed no interest in planning for babies. However, during the pandemic, 100 of the 268 abandoned their plans for fear of becoming infected (*n* = 28), fear of the consequences of pregnancy (*n* = 58), and fear of economic difficulties (*n* = 58). In contrast, 140 among the 1,214 participants indicated that they were now planning to have children during the pandemic. The reasons for such changes in opinion included having more free time (*n* = 36), increased couple interactions (*n* = 26), wishing to bring about some changes in the couples' lives (*n* = 70), and need for positivity (*n* = 56). Notwithstanding, there was an overall reduction in frequency of sexual intercourse during the pandemic (14). In another study (*n* = 2,009) carried out by the Guttmacher Institute in the USA, 40% women reported having changed their plan to not have a child during pandemic (16). The study also reported that lower-income women (36%) were more likely to have trouble and delays in having access to contraception and birth controls than higher-income women (31%) during the pandemic.

### The Impact of Anti-COVID-19 Measures on Fertility Rates in LMICs

If we re-examine the consequences of a pandemic on TRF, although a baby boom, i.e., a remarkable sudden increase in the birth rates when compared to normal rates, is unlikely to occur in western countries despite economic problems, psychological distress, household stress, and shortage of health services, controlling the anticipated baby boom is impracticable among individuals in low-middle income countries (LMIC). Moreover, in LMICs (e.g., India and Bangladesh), the impact of COVID-19 on fertility appears to be quite different. While socioeconomic factors are intimately related to risk awareness as related to pregnancy during a pandemic, and therefore highly educated sectors of the population in LMIC are unlikely to plan family expansion during this situation, some cases of conception may still occur (12). For instance, prolonged lockdown may result in a large number of women or men not having access to various forms of contraception, also a major determinant of baby booms after an epidemic has occurred. The Ipas Development Foundation, which focuses on contraception and abortion in India, an LMIC of Southeast Asia, estimated that about 1.85 million women were unable to gain access to abortions between March and May 2020 (17). Another organization in the same country, Reproductive Health Service reported that about 25 million people were unable to access contraception in May 2020 during lockdown. Among all other less privileged sectors of the population in LMIC, the lack of access to birth control services is further apparent, and likely to result in millions of unintended pregnancies, unsafe abortions, and maternal deaths (18). Moreover, during the lockdown women are not able to go to the clinics for their regular check-ups and pregnancy tests, and consequently, they are not always able to prevent unintended pregnancies. Additionally, the practice of family planning is comparatively low among illiterate individuals due to poverty and lack of education and resources in LMICs (19). Such individuals do not have clear concepts and awareness about proper spacing between pregnancies, usage of condoms, and of female contraceptive methods (20, 21). As a result, unintended pregnancies are unlikely to be reduced among this group. Due to the lockdown, individuals are in their houses with their partners and because of job losses or interrupted work-related activities, the increased time spent at home will further escalate the possibility of a baby boom in rural areas during this pandemic.

### The Possible Direct Impacts of COVID-19 on Fertility Rates

Few unresolved or poorly understood factors could also significantly affect the fertility rate during any pathogenic outbreak. Given the 1918 influenza pandemic as an example, pregnant women were the hardest hit among all infected individuals, but the reason behind such observations are still a subject of debate (22–24). Since many factors regarding COVID-19 remain still poorly understood, the chance of direct impact of SARS-CoV-2 (the causative agent of COVID-19) on both male and female fertility cannot be excluded. SARS-CoV-2 binds to the Angiotensin Converting Enzyme-2 (ACE-2) receptors to enter the cells of human body. Several hypotheses have pointed the presence of ACE-2 receptors on male Leydig cells and female ovaries as the possible thread to directly affect human fertility (25, 26). Therefore, how differently COVID-19 affects male and female fertility *per se* is not yet clear, and as a result, the actual impact of SARS-CoV-2 on overall fertility cannot entirely be incorporated into accurate estimates of future TFR (20, 21). In addition, the knowledge gap about the vertical transmission of the SARS-CoV-2 virus, along with the inability to universally diagnose asymptomatic patients remains another concern to consider the direct impact of COVID-19 pandemic on fertility rate (27). If SARS-CoV-2 is vertically transmitted from asymptomatic mother to child, the assumption on COVID-19 not affecting pregnancy outcomes or birth rates may be misleading. Furthermore, embryology laboratory personnel who is infected and asymptomatic can contaminate the gamete/embryo during manipulation required for *in vitro* fertilization (IVF) and thus can affect the fertility rate unknowingly. These unknown factors and certainly many others that remain unaccounted may influence professional societies recommendations (10), as well as lead to public opinion shifts regarding pregnancy decisions.

### The Impacts of COVID-19-related Morbidity and Mortality on Fertility Rates

Additionally, the death rate of COVID-19 may adversely impact TFR; however, considering that COVID-19 mortality rates are particularly elevated among older individuals and those with underlying chronic disease, the overall direct effect of mortality is likely to be minor on TFR. We should point out that historical evidence from high mortality events such as wars, diseases, famines, heatwaves, and storms typically have an immediate negative effect on fertility rates, whereby mortality affects fertility by both a replacement effect and a hoarding effect. By replacement (or volitional) effect, we indicate compensation for birth loss (i.e., a response by couples to plan for a new baby because they have lost one), especially evidenced in societies where extended families living together are the norm, and where children are valuable for their support in their parents' old age and for their economic contribution to the family. A significant rise in stillbirths was observed during the ongoing pandemic in UK, India and Nepal and the study carried out among 20 thousand women in nine hospitals across Nepal revealed about 50% increase in stillbirth rates due to inaccessibility to health facilities and antenatal support (28). This suggests that the global fertility rate could be also influenced by replacement effect more specifically in developing countries. By hoarding effect, we signify expected mortality risk of offspring by their parents (i.e., a response by couples to expected mortality of their offspring which causes them to plan for more babies) (29, 30).

## Correspondence Between COVID-19 and Other Preceding Epidemics

A large number of deadly disasters have previously occurred in the world history. From influenza epidemics to COVID-19, all have taken hundreds of thousands of lives. Studies have shown that such high fatality disasters lead to a decline in births in the several months that follow such events. The Great Finnish Famine (1866–1868) killed more than 0.2 million people in Finland (i.e., 10% of the country population). The birth rate during the epidemic was lower compared to the period 1801–1850 (31). The birth rate later markedly increased shortly after the famine ended. The Spanish flu (1918) is the most destructive flu pandemic in modern history and killed 50 million people worldwide. A unique feature of this virus was the high death rate among young adults aged 20–40 years (32). There were no significant changes noticed in fertility rates between 1913 and 1918, and the fertility rate was at its lowest in 1919. However, in 1920, a baby boom occurred in European countries including Norway, Sweden, and the UK. This surge in natality was identified as reflecting the tendency of many couples rushing to wed and then conceive children after surviving the epidemic (33, 34). A surge in fertility rate was also observed 9–12 months after the Great Kanto Earthquake in Japan (1923). Experts suggested that victims of the disaster sought motherhood because of child loss in the earthquake (35).

More recently, studies suggest that fertility rates also declined during the Severe Acute Respiratory Syndrome (SARS) epidemic (2003) and the Zika virus outbreak (2015–16). In a study carried out in Taiwan that compared to pre-SARS period, the market share for childbirth health services dropped in medical centers (5.2%) and regional hospitals (4.1%) with reduced cesarean rates during the peak SARS period (36). The Ebola epidemic in Africa killed 50% of infected individuals, spread by means of bodily fluids, and had a case fatality rate up to 70%. During and after the Ebola epidemic, the birth rate declined, but after the announcement of several countries as being Ebola-free, the birth rate temporarily rose. For instance, in Liberia, a sharp decline in birth rates was observed during the first 6 months from the beginning of the Ebola outbreak, whereas a 33% rise was reported for 5 months in the 17 months preceding the outbreak (37, 37, 38).

**Figure 1:**
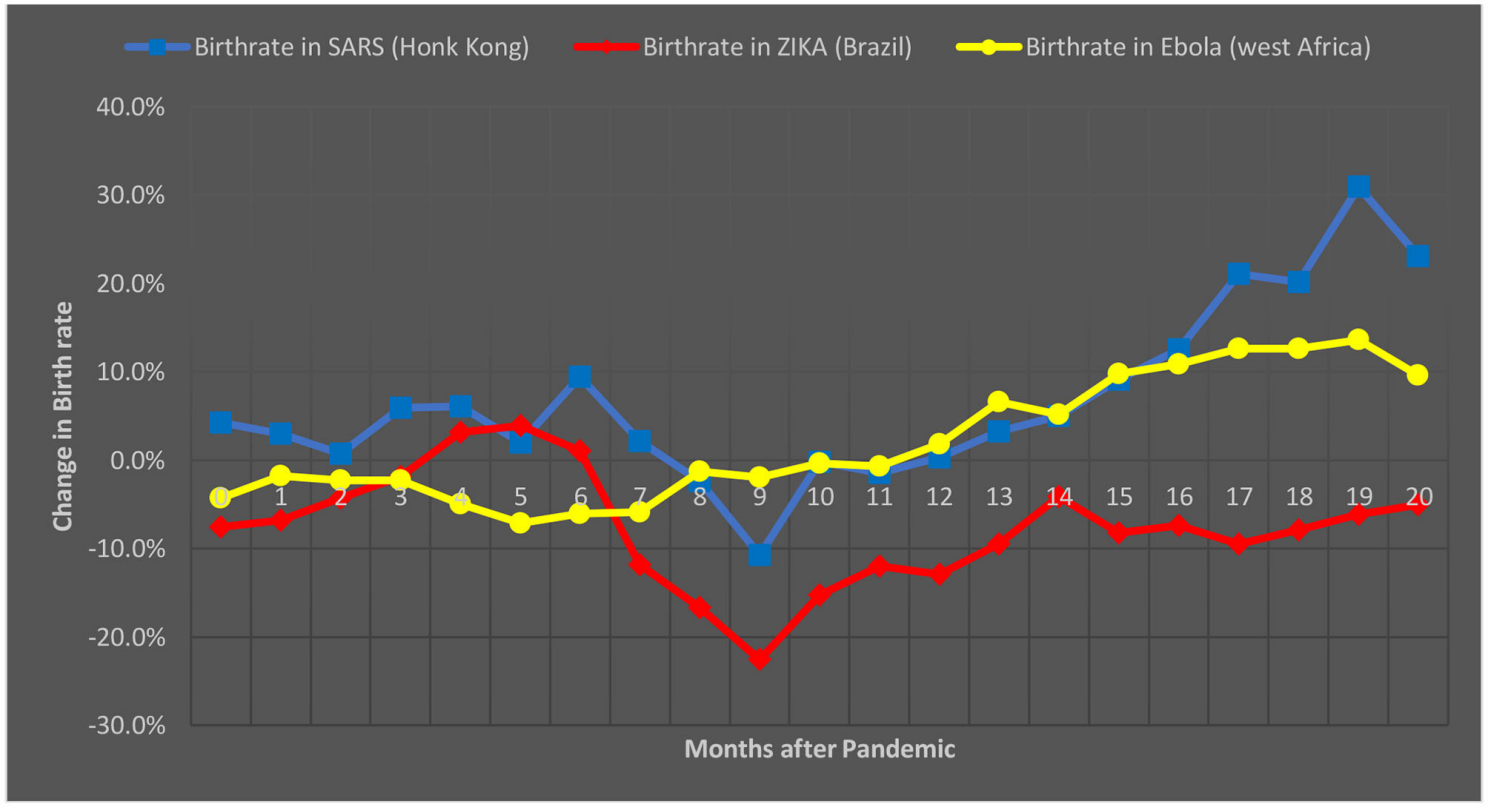
Change in birth-rate in months after the start of three recent epidemics, namely SARS, Zika, and Ebola (Source: The Economist; Institute for Family Studies).

In Figure 1, we depict monthly fluctuation (percent change in monthly birth rate after outbreak) from the start of the SARS, Zika, and Ebola epidemics in Hong Kong (2002), Brazil (2015), and West Africa (2016). After several months (8–12 months) of the epidemics, a reduction in birth rates was apparent and was followed by a noticeable upward trend in the birth rates that lasted well into 20 months after the beginning of each of these epidemics.

Overall, these observations indicate that during all these three recent epidemics, the birth rates decline immediately after the epidemic and recover or further surpass pre-epidemic levels within a year and thereafter. The reclamation of fertility took place mainly because of the replacement effect and the hoarding effect. The loss of family members, relatives, or friends appeared to result in replacement fertility. Additionally, the fear of existence and insecurity influenced the hoarding effect (39). The baby boom after the Spanish flu pandemic was because many women had experienced mortality directly (i.e., loss in their own family) or indirectly (observed death in neighborhood or community). Therefore, the birth rates vary during epidemics and pandemics in different regions because of the different factors influencing birth rates.

## Discussion

A recent demographic study estimated that the total number of COVID-19 infections is four times larger than the number of confirmed cases. As mentioned, if SARS-CoV-2 exerts a direct effect on either male or female fertility, the impact of such asymptomatic infections on birth rates could be augmented, and yet unless universal testing is instituted for detection of all asymptomatic cases, the attributable factor to such decline in fertility and consequently birth rates would not be identified. At present, and with very limited evidence, it is somewhat difficult to predict whether and how COVID-19 will affect birth rates. However, considering factors such as changes in socio-economic conditions, mental health, mortality rates, and direct effects of the virus on fertility, and incorporating lessons learned from the previous pandemics, it would be reasonable to postulate that the COVID-19 pandemic may significantly affect future birth rates with long-term effects.

The aforementioned cross-sectional study in Italy, reported that 37.3% had abandoned intentions of having a baby due to the future economic climate, but also that 4.3% had tried to achieve pregnancy (9). Therefore, this change to family planning will to some extent mitigate each other, and the birth rate after 9 months (Bertillon effect) will not be as pronounced due to the counterbalancing effects of these two factors. After an initial reduction, it is expected that birth rates will rise again due to the aforementioned mortality replacement and hoarding effects. However, more precise estimates of the birth rates are unknown because previous studies of epidemics suggest a range from 0.25 to 2 births being added per each death toll in the course of 1 to 5 years after an epidemic. The reduction of 1 birth in 1918 during Spanish flu, was followed by an increase of 1.5 conception 1 year later and resulted in a baby boom (32, 37). This suggests that the COVID-19 pandemic is also very likely to influence the global fertility rate significantly.

The economic recession seems to be a major regulator for affecting the birth rates differently across countries with different socioeconomic settings. Moreover, since every country has unique characteristics in terms of literacy, family planning, rate of disease spread, mortality, and morbidity, different trajectories in fertility and birth rates are anticipated. Again, the countries will start recovering their normal economic state after the pandemic has abated which will also differ from one country to another. The recovery period thus may also significantly influence the global fertility rates for long term in different manners across HICs and LMICs.

Furthermore, the availability of contraception and health care facilities during the pandemic appear to affect the fertility rates greatly in LMICs than HICs. Therefore, a short-lasting drop followed by a later sudden rise in birth rates due to the pandemic is expected to normalize rapidly in developed economies, while a much more variable pattern should emerge in LMICs. And thus, governments in LMICs should ensure the emergency supports to avoid unintended baby boom in such countries during ongoing pandemic. The health agencies should monitor and record the factors associated with undesired events like stillbirth and prevent these from happening again. Moreover, lessons learned from the COVID-19 pandemic should be executed to avoid any future similar circumstances caused by outbreaks or other natural disasters.

## Conclusion

The COVID-19-related pandemic is negatively impacting human welfare in many domains, and as a result, birth rates are likely to be affected, albeit differentially in developed economies and in LMICs. While initial reductions in birth rates are likely, it is overall expected that a rebound of such rates will take place. Consequently, LMIC governments can play an important role in preventing undesirable baby booms by implementing measures, such as ensuring continued access to family planning centers, and instituting informative public education campaigns.

## Data Availability Statement

The original contributions generated for the study are included in the article/supplementary materials, further inquiries can be directed to the corresponding author/s.

## Author Contributions

MU and DG conducted the complementary literature searches and reviews. MU, AB, and YA wrote the initial draft of the manuscript. YA conceived the study design. MG and DG edited and revised the manuscript. All authors approved the final manuscript.

## Conflict of Interest

The authors declare that the research was conducted in the absence of any commercial or financial relationships that could be construed as a potential conflict of interest.
